# Propofol inhibits colon cancer cell stemness and epithelial-mesenchymal transition by regulating SIRT1, Wnt/β-catenin and PI3K/AKT/mTOR signaling pathways

**DOI:** 10.1007/s12672-023-00734-y

**Published:** 2023-07-25

**Authors:** Runjia Wang, Shuai Li, Qi Hou, Bo Zhang, Huaqing Chu, Yu Hou, Cheng Ni, Li Sun, Yuliang Ran, Hui Zheng

**Affiliations:** 1 Department of Anesthesiology, National Cancer Center/National Clinical Research Center for Cancer/Cancer Hospital, Chinese Academy of Medical Sciences and Peking Union Medical College, Beijing, 100021 China; 2 State Key Laboratory of Molecular Oncology, National Cancer Center/National Clinical Research Center for Cancer/Cancer Hospital, Chinese Academy of Medical Sciences and Peking Union Medical College, Beijing, 100021 China; 3 Department of Anesthesiology, National Cancer Center, National Cancer Clinical Research Center, Shenzhen Cancer Hospital, Chinese Academy of Medical Sciences and Peking Union Medical College, Shenzhen, 518116 China

**Keywords:** Propofol, SIRT1, Stemness, Epithelial-mesenchymal transition, Colon cancer

## Abstract

**Background:**

Propofol is a common sedative-hypnotic drug used for general anesthesia. Recent studies have drawn attention to the antitumor effects of propofol, but the potential mechanism by which propofol suppresses colon cancer stemness and epithelial-mesenchymal transition (EMT) has not been fully elucidated.

**Methods:**

For the in vitro experiments, we used propofol to treat LOVO and SW480 cells and Cell Counting Kit-8 (CCK-8) to detect proliferation. Self-renewal capacity, cell invasion and migration, flow cytometry analysis, qPCR and Western blotting were performed to detect the suppression of propofol to colon cancer cells and the underlying mechanism. Tumorigenicity and immunohistochemistry experiments were performed to confirm the role of propofol in vivo.

**Result:**

We observed that propofol could suppressed stem cell-like characteristics and EMT-related behaviors, including self-renewal capacity, cell invasion and migration in colon cancer cells, and even suppressed tumorigenicity in vivo. Furthermore, investigations of the underlying mechanism revealed that propofol treatment downregulated SIRT1. SIRT1 overexpression or knockdown affected the stemness and EMT of colon cancer cells. Additionally, propofol reversed stemness and EMT in cells with overexpressing SIRT1 and subsequently inhibited the Wnt/β-catenin and PI3K/AKT/mTOR signaling pathways. Wnt/β-catenin pathway inhibitor and PI3K/AKT/mTOR pathway inhibitor blocked the propofol-induced reduction of sphere-formation and cell invasion-migration.

**Conclusion:**

Propofol inhibits LOVO and SW480 cell stemness and EMT by regulating SIRT1 and the Wnt/β-catenin and PI3K/AKT/mTOR signaling pathways. Our findings indicate that propofol inhibits SIRT1 in cancer and is advantageous in colon cancer surgical treatment of patients with high SIRT1 expression.

**Supplementary Information:**

The online version contains supplementary material available at 10.1007/s12672-023-00734-y.

## Introduction

Colon cancer is the second most prevalent malignant neoplasm [1] and the second most deadly cancer in the world [2], with higher rates of cancer recurrence or metastasis at final stages [3]. The 5-year survival rate of colon cancer patients is low because of cancer chemotherapy resistance, aggressiveness, metastasis, and relapse. This leads to patients’ demand for surgery under anesthesia during cancer treatment. As a general anesthesia drug, propofol has become the most commonly used intravenous anesthetic in modern anesthesia [4], and it is widely used in tumor treatment and surgery [4]. In recent years, the effects of general anesthetics on tumor cells and the tumor microenvironment have attracted much attention. Propofol has antitumor potential [4], and patients administered propofol-based total intravenous anesthesia (TIVA) have better overall survival than those administered inhalation anesthesia during cancer surgery [5]. Cancer cell studies in vitro have found that propofol may suppress the cancer cell invasion and proliferation and promote the apoptosis [6] of pancreatic cancer cells [7], colon carcinoma cells [8], gastric cancer cells [9], glioma cells [10], lung adenocarcinoma cells [11], and breast cancer cells [12], etc. Although propofol has been proven to be reliable in general anesthesia, its influence on the stemness and epithelial-mesenchymal transition (EMT) of colon cancer cells has yet to be elucidated.

Cancer stem cells (CSCs) demonstrate pluripotent differentiation ability, are characterized by their self-renewal ability, and they have been verified to contribute to cancer metastasis, drug resistance, and recurrence [13]. CSCs are present in many types of cancers, such as colon cancer [14], gastric cancers [15], and breast cancer [16]. Stemness is the ability of normal or transformed cells to self-renew and produce differentiated offspring [17]. Once cancer cells acquire stemness features, they express specific stemness markers which are essential for the maintenance of pluripotency and self-renewal [18]. EMT includes dynamic changes in cellular organization during the transition from an epithelial to a mesenchymal phenotype, which results in functional changes in cell migration and invasion [19]. EMT occurs normally during early embryonic development, to enable a variety of morphogenetic events, as well as later in development and during wound healing in adults [19]. It is known to be activated during cancer pathogenesis, tissue fibrosis [19] and stemness [20]. EMT induces stemness in many cancer types, including cancers of the mammary gland, lung, colon, prostate, and ovary [20]. However, whether propofol can alter stemness and EMT in colon cancer cells remains unknown.

SIRT1 is a nicotinamide adenine dinucleotide (NAD^+^)-dependent class III protein deacetylase [21]. SIRT1 participates in numerous cellular processes by deacetylating specific substrates and plays an essential role in tumorigenesis [21]. SIRT1 is upregulated in colorectal cancer samples [3], and clinical analysis indicated a vital association between high SIRT1 expression and poor outcome in colorectal-cancer patients [22]. SIRT1 is significant in maintaining the characteristics of colon CSCs [21], promoting EMT, and metastasis in colon cancer cells [23] and their stemness [3]. However, whether propofol could regulate the SIRT1 level in cancer further influence stemness and EMT in colon cancer still needs to be further investigated.

Importantly, although propofol has been demonstrated to be associated with the occurrence and progression of cancer, far less is learned about the role of propofol in colon CSCs. In the present study, we investigated the relationship between propofol and the stem cell-like characteristics of colon cancer cells and found that propofol inhibited the stemness and EMT of LOVO and SW480 cells. In addition, propofol downregulated SIRT1 in cancer and inhibited the Wnt/β-catenin pathway and PI3K/AKT/mTOR pathway. Furthermore, the in vivo results showed that propofol inhibited tumor proliferation.

## Materials and methods

### Cell culture and reagents

The human colon cancer cell lines LOVO and SW480 were obtained from the Chinese Academy of Medical Sciences. Cell lines were cultured in DMEM cell culture medium (Livning, China) supplemented with 10% fetal bovine serum (Kangyuan Biotech, China) containing 1% penicillin–streptomycin at 37 °C in a humidified atmosphere with 5% CO_2_. Propofol (MCE, USA) was dissolved in dimethyl sulfoxide (DMSO, Sigma-Aldrich, USA) and supplied in the culture medium. The final dose of DMSO was < 0.1%. Then, LOVO and SW480 cells were treated with 2.5 µg/mL, 5 µg/mL, and 10 µg/mL propofol for 0,6,12 and 24 h. The control groups with0 µg/mL propofol were treated with the same volume of DMSO for 0, 6, 12 and 24 h.

### Cell proliferation assay

Cells were seeded in 96-well plates (3000 cells/well) and cultured for 24 h. Then the cells were treated with distinct concentrations of propofol for distinct hours. Cell Counting Kit-8 (CCK8, Dojindo Chemistry, Japan) reagent was added to every well and the cells needed to be further cultured for 2 h. The optical density at 450 nm was detected by a microplate reader (Bio-Rad, USA). By measuring the optical density of each well, the cells viability curve was determined, and the results were average of 3 wells.

### Self-renewal assay

We used spheroid-formation experiments to explore the self-renewal ability of colon cells. The cells were seeded in 24-well ultra-low attachment plates (Corning, USA) about 500 cells/well and cultured in serum-free DMEM/F12 medium supplemented with 0.8% methylcellulose (Sigma-Aldrich, USA), 10 ng/mL LIF, 20 ng/mL EGF, B27(1:50), and 20 ng/mL bFGF. The cells were cultured at 37 °C in 5% CO_2_ for 14 days. Then the spheroids were counted under a microscope.

### Western blot and antibodies

Western blot and gray-scale value measuring were performed as described before [3]. Twenty micrograms of protein separated from each sample was separated using 10% SDS-PAGE and electrotransferred to a PVDF membrane (Millipore, USA). Membranes were blocked at room temperature for 1 h, incubated with primary antibodies overnight at 4 °C and then incubated with secondary antibodies. The membranes were washed and detected using an enhanced chemiluminescence (ECL) reagent kit (Livning, China) with Image Quant LAS 4000 CCD camera (GE Healthcare, USA).The catalogue number of the antibody is provided in the supplement document.

### Flow cytometry analysis

Flow cytometry was performed as described elsewhere [3]. Cells were stained with anti-CD24 and anti-CD44 primary antibodies (BD Pharmingen) for 1 h. After washing with PBS three times, the cells were stained with secondary antibody..Then incubated at room temperature for 1 h in dark. Cells were washed and then corrected optical path before flow cytometry analysis with Attune NxT, Thermo Fisher.

### Transwell™ invasion and migration assay

A total of 1 × 10^5^ serum-starved cells were resuspended in 200 μL serum free medium and plated in the top of a Transwell™ chamber (24-well insert; pore size, 8 μm; Corning). Diluted Matrigel (BD Biosciences, USA) was coated on invasion assay. After 24 h, the number of infiltrating cells was counted by a light microscope, and invasion and migration of cells were analyzed quantitatively.

### Quantitative PCR

Total RNA was extracted using TRIzol (Servicebio, China). The RNA was reversely transcribed into cDNA using the Servicebio®RT First Strand cDNA Synthesis Kit (Servicebio, China) with genomic DNA eraser. The expression levels of target genes and GAPDH were evaluated by quantitative PCR. The sequences for PCR primers are provided in the supplemental document. We used the 2^−ΔΔCt^ method to calculated changes in transcript abundance of tested genes.

### siRNA and plasmid DNA transfection

LOVO and SW480 cells (1 × 10^5^) were seeded onto 6-well plates. Short interfering RNA of SIRT1 (siSIRT1) 5′- CCAAGCAGCUAAGAGUAAUTT-3′ (sense), 5′- AUUACUCUUAGCUGCUUGGTT-3′ (antisense) (Genepharma, China) and pcDNA-SIRT1 (Genepharma, China) were transiently transfected into LOVO and SW480 cells when cells reached 70–80% confluence using Lipofectamine™ 3000 Reagent (Invitrogen, USA). After 48 h transfection, the cells were collected for further experiments.

### Tumorigenicity analysis in BALB/c nude mice and immunohistochemistry (IHC)

Animal study was approved by Animal Ethics Review Committee of Cancer Hospital, Chinese Academy of Medical Sciences (No.NCC2022A302). BALB/c nude mice (4–5 weeks old) were bought from HFK Bioscience Company (Beijing, China). 5 × 10^6^ SW480 or 2.5 × 10^6^ LOVO cells were subcutaneously injected into the backs of mice (5 mice/group). Seven days later, propofol (45 mg/kg) was injected every 2 days by intraperitoneal. The tumor size was measured every 2 days. All mice were sacrificed on day 30 after inoculation. The tumor weight of each mouse was measured, too. Immunohistochemistry staining was performed as described elsewhere [24]. Histoscore (H-score) was calculated by a semi-quantitative assessment of both the percentage and the intensity of staining. Four visual fields were randomly selected for each section, and scores of staining intensity and percentage of positive cells were performed. The score of staining intensity multiply by score of percentage of positive cells is the final score. The scoring was performed by two independent pathologists. Primary antibodies were provided in the Additional file 1.

### Statistical analysis

All data are shown as the mean ± standard deviation (SD) derived from at least three independent tests. The statistical significance was assessed by unpaired Student’s t tests and results were significantly if P < 0.05. SPSS 26.0 and GraphPad Prism 8.0 were used to perform all analyses.

### Bioinformatic analysis by the UCSC Xena platform

Bioinformatic analyses were performed according to The Cancer Genome Atlas (TCGA) TARGET GTEx cohort in the UCSC Xena platform [25]. Only colon samples were selected and included for further analysis of the RNA expression of SIRT1. The expression levels of SIRT1 between adenocarcinoma and normal tissues were compared by statistical analysis. The correlation between the expression levels of SIRT1 and Nanog and ALDH1A1 in colon samples was analyzed, too. Website: https://xena.ucsc.edu/.

## Results

### Propofol inhibits LOVO and SW480 cell proliferation

**Fig. 1:**
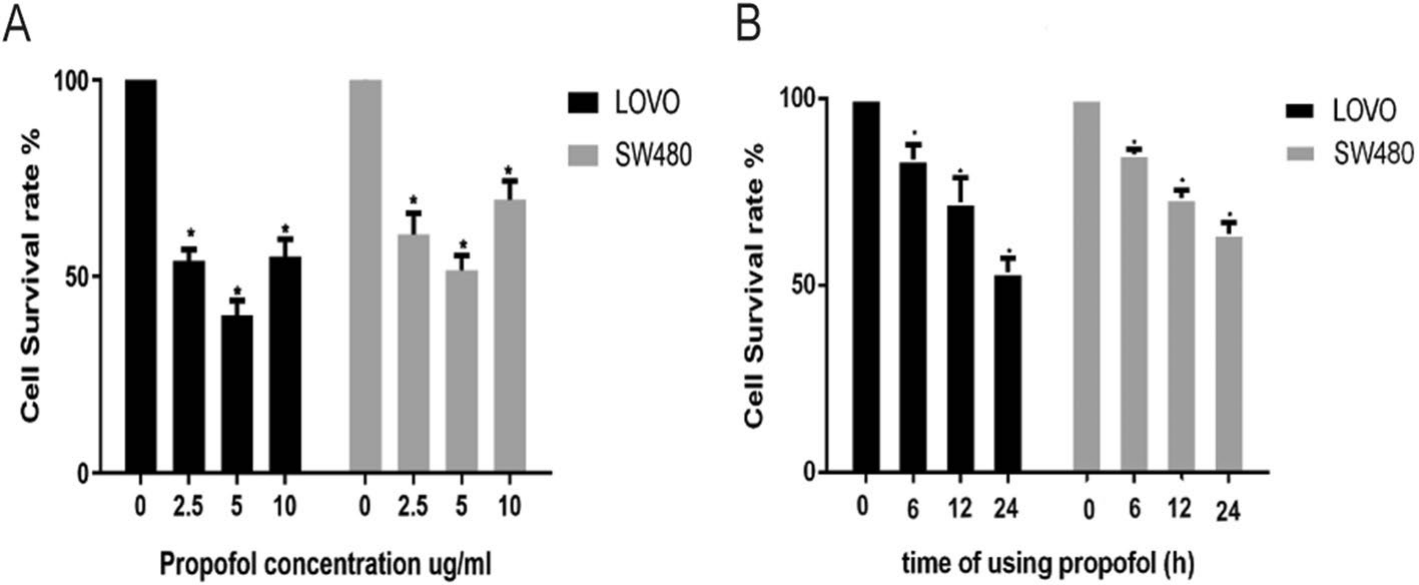
Propofol Inhibits LOVO and SW480 Cell Proliferation. Cell lines were treated with propofol for 24 h at concentrations of 0 µg/mL, 2.5 µg/mL, 5 µg/mL and 10 µg/mL, and the inhibitory effect of 5 µg/mL propofol on cells was tested for different times (0 h, 6 h, 12 h, and 24 h). P < 0.05

Cell lines were treated with propofol for 24 h at concentrations of 0 µg/mL, 2.5 µg/mL, 5 µg/mL and 10 µg/mL. The results showed that propofol could decreased the survival rate of colon cancer cells (Fig. 1A). Then, we assessed the inhibitory effect of 5 µg/mL propofol on cells after different exposure times (0 h, 6 h, 12 h, and 24 h), and the results showed that propofol could decrease the survival rate of colon cancer cells in a time dependent manner (Fig. 1B). We treated cell lines with 5 µg/mL propofol for 24 h for follow-up experiments.

### Propofol inhibits the stemness of colon cancer cells and the Wnt/β-catenin pathway in vitro and in vivo

**Fig. 2:**
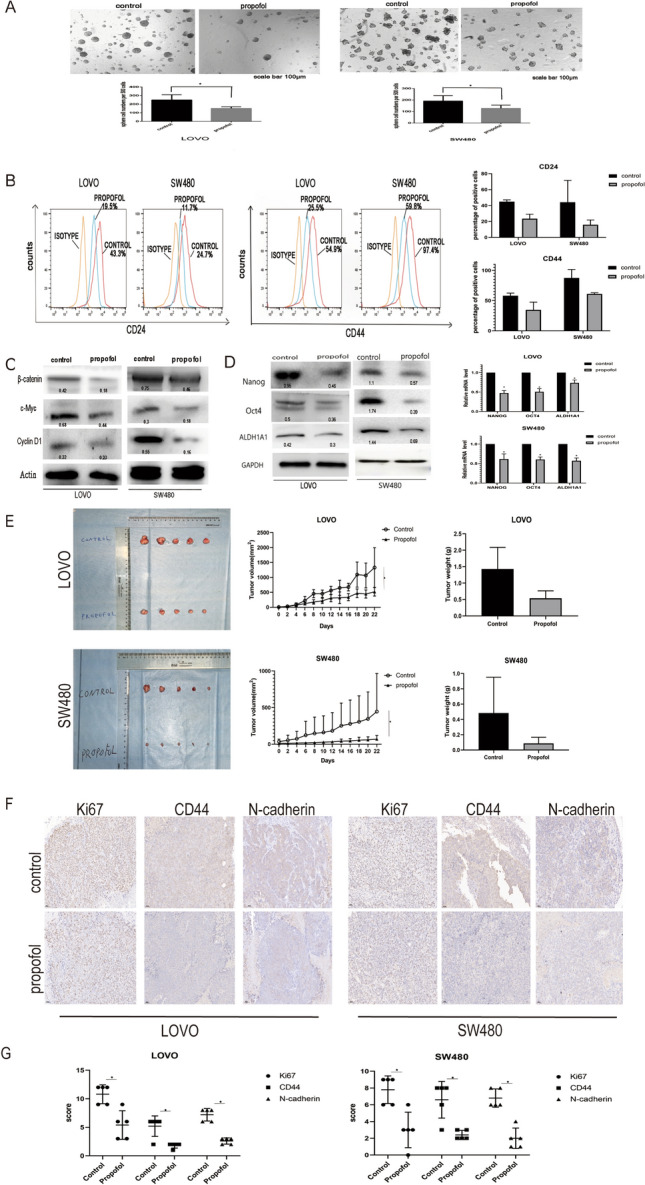
Propofol inhibits the stemness of colon cancer cells and Wnt/β-catenin pathway in vitro and in vivo. **A** Analysis of the self-renewal abilities of LOVO and SW480 cells after treatment with or without propofol. Scale bar = 100 μm. **B** The expression of CD24 and CD44 in LOVO and SW480 cells was measured by flow cytometry analysis. **C** Western blot analysis for expression of Wnt/β-catenin signaling pathway. **D** Western blot analysis and qPCR after treatment with or without propofol. **E** The in vivo effect of propofol treatment was evaluated in xenograft mouse models. Tumour volume and weight were measured for each mouse. **F** IHC detection of Ki‐67, N‐cadherin and CD44 is shown. **G,** **H**-score of IHC. Scale bar = 50 μm. *P < 0.05

To analyze the role of propofol on stem-like characteristics in colon cancer cells, we performed sphere formation assay in LOVO and SW480 cells under serum-free and non-adherent conditions with 5 µg/mL propofol treatment. The results showed that propofol induced a significant decrease in the number of tumor-spheres (P < 0.05) (Fig. 2A). Then, we used flow cytometry analysis to analyze the proportion of cells expressing stem cell markers CD24 and CD44 in different cell lines with or without propofol treatment, and the results showed that propofol decreased the proportion of cells expressing stem cell markers (Fig. 2B). We also examined the expression of stemness-related markers by Western blotting and qPCR. As shown in Fig. 2D, propofol decreased RNA and protein expression of Nanog, ALDH1A1 and Oct4. Additionally, propofol treatment significantly decreased the protein expression of β-catenin, cyclin D1, and c-Myc (Fig. 2C).

To confirm the in vitro results, cell lines were used to establish mouse xenograft tumor models. Tumor volume and weight were analyzed in the control and propofol treatment groups. However, as shown in Fig. 2E, propofol treatment significantly decreased the volume and weight of tumor xenografts. Thereafter, IHC staining confirmed that Ki67, CD44 and N-cadherin were downregulated with propofol treatment (Fig. 2F, G), which was consistent with the findings from the in vitro results. Collectively, these results suggest that propofol inhibits the stemness of colon cancer cells and the Wnt/β-catenin pathway.

### Propofol inhibits the EMT of colon cancer cells and the PI3K/AKT/mTOR signaling pathway

**Fig. 3:**
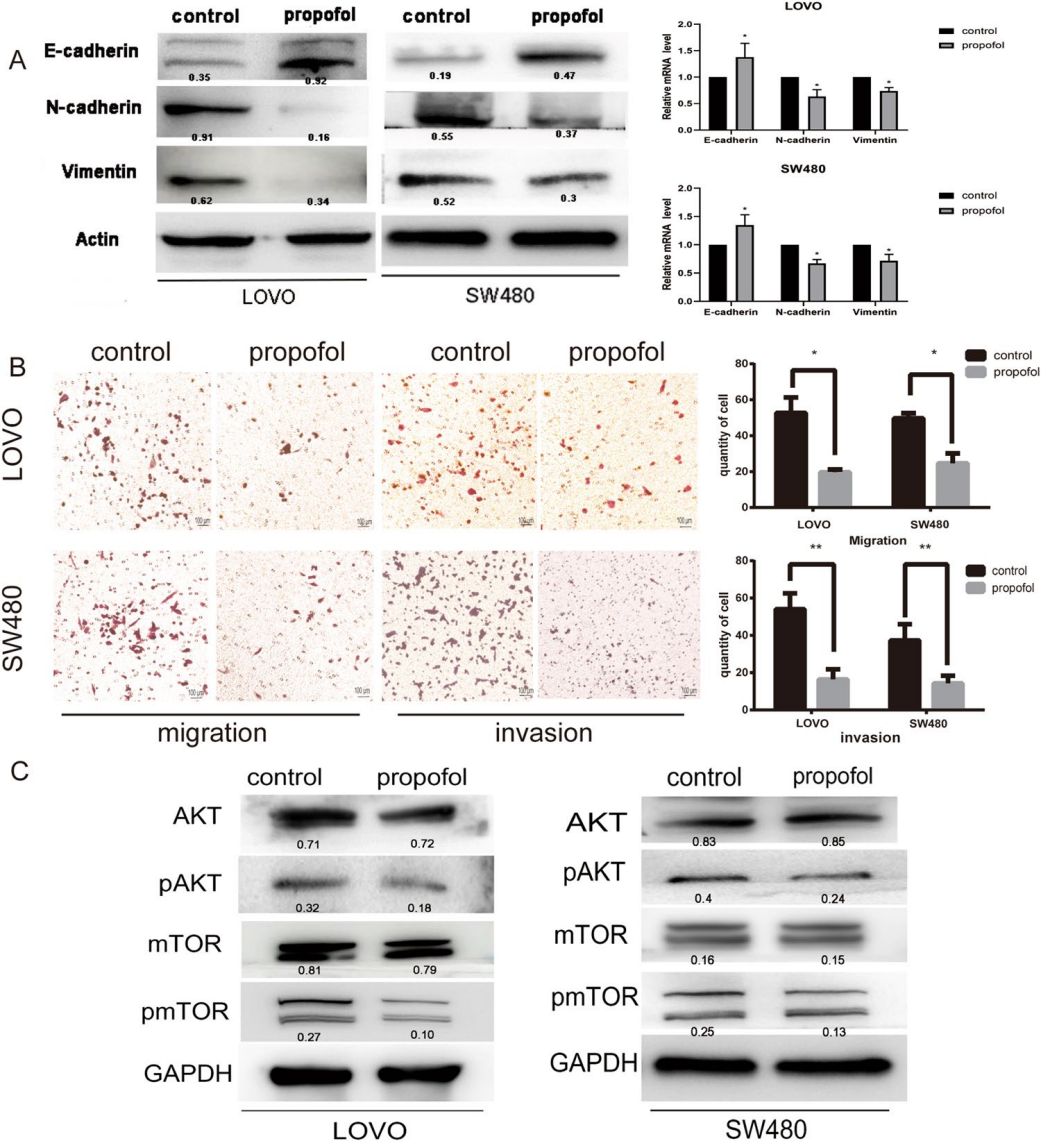
Propofol inhibits EMT of colon cancer cells and PI3K/AKT/mTOR signaling pathway. **A** Western blot analysis and qPCR of EMT-related markers expression of after treatment with or without propofol. **B** After treatment with propofol, the invasion and migration abilities of LOVO and SW480 cells were inspected by and Transwell assays. Scale bar = 100 μm **C** Western blot analysis of the expression of PI3K/AKT/mTOR signaling pathway after treatment with or without propofol. *P < 0.05

To determine whether propofol plays a role in EMT, LOVO and SW480 cells were treated with 5 µg/mL propofol for 24 h. The expression of the EMT markers N-cadherin, E-cadherin and Vimentin was assessed by Western blotting and qPCR. As shown in Fig. 3A, propofol significantly decreased the expression of N-cadherin and Vimentin, whereas increased the expression of E-cadherin. Furthermore, the number of migrated cells was decreased in the propofol treated group compared with the control group, confirming the inhibitory effect of propofol on EMT (Fig. 3B). Additionally, propofol treatment significantly decreased the expression of AKT, p-AKT, mTOR and p-mTOR at the protein level (Fig. 3C). These results indicate that propofol inhibits EMT and the PI3K/AKT/mTOR pathway in colon cancer cells.

### Propofol down-regulates SIRT1, and SIRT1 is involved in stemness and EMT through the Wnt/β-catenin signaling pathway and PI3K/AKT/mTOR signaling pathway

**Fig. 4:**
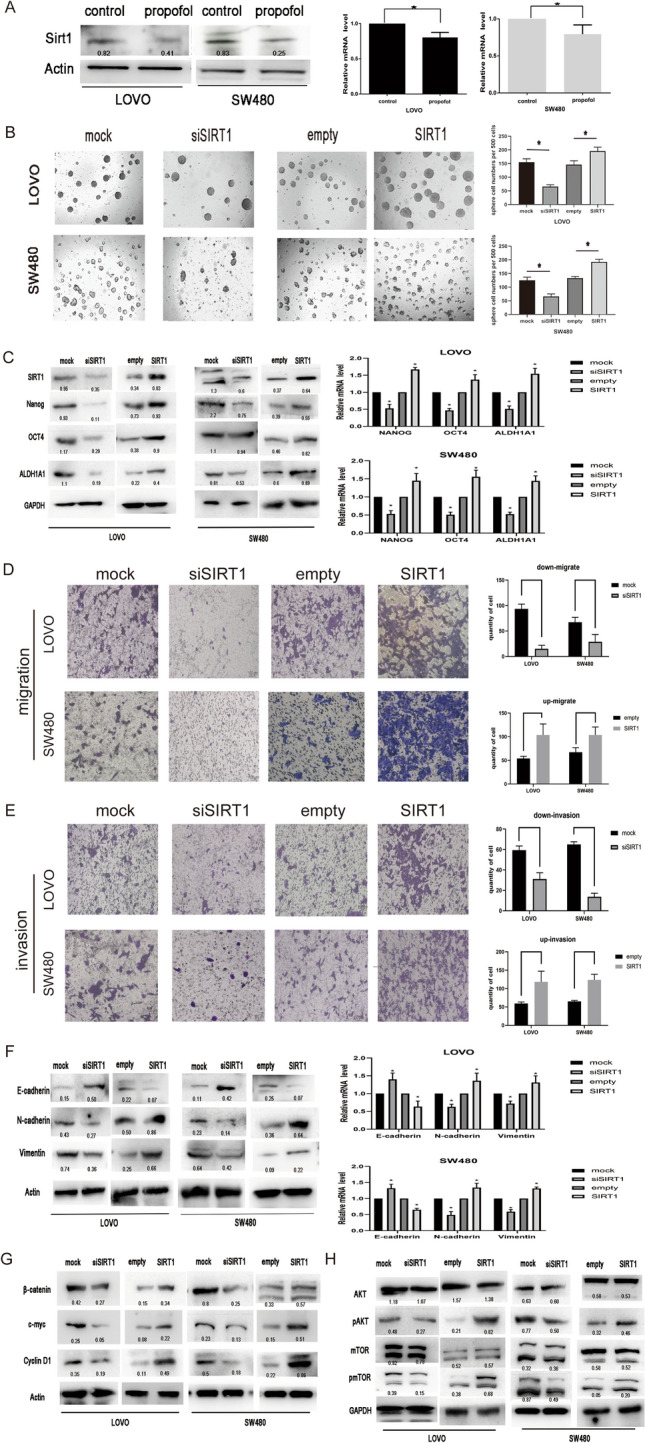
Propofol down-regulates SIRT1 expression, and SIRT1 is involved in stemness and EMT through Wnt/β-catenin signaling pathway and PI3K/AKT/mTOR signaling pathway. **A** Level of SIRT1 in LOVO and SW480 cells after propofol treatment measured by Western blot analysis and qPCR. **B** Analysis of the self-renewal abilities of LOVO and SW480 cells expressing mock, siSIRT1, empty and plasmid DNA SIRT1. Scale bar = 100 μm. **C** Expression of stem cell markers in LOVO and SW480 cells was detected by Western blot and qPCR. **D** and **E**. The invasion and migration ability of LOVO and SW480 cells were inspected by Transwell assays after downregulated and upregulated SIRT1. Scale bar = 100 μm. **F** Expression of EMT markers was detected by Western blot and qPCR. **G, H** Western blot of PI3K/AKT/mTOR signaling pathway and Wnt/β-catenin signaling pathway in LOVO and SW480 cells *P < 0.05

To explore whether SIRT1 is regulated by propofol, colon cancer cells were treated with 5 µg/mL propofol for 24 h. The results indicated that propofol decreased SIRT1 gene and protein expression compared with that in the control group according to qPCR and Western blotting (Fig. 4A).

To explore the impact of SIRT1 on stemness and EMT, we first used siRNA and plasmid DNA to knockdown and overexpress SIRT1 in LOVO and SW480 cells, and confirmed these perturbations by Western blotting (Fig. 4C). We investigated the impact of SIRT1 on the capacity of self-renewal. Upon SIRT1 overexpression, the self-renewal ability of LOVO and SW480 cells was significantly increased (Fig. 4B). In contrast, the self-renewal capacity of SIRT1 knockdown cells was markedly decreased compared to that of control cells. Moreover, we found that the expression of the stem cell markers Nanog, Oct4, and ALDH1A1 increased in SIRT1-overexpressing cells, while the expression of these markers was decreased in SIRT1-knockdown cells (Fig. 4C). Taken together, these results suggest that SIRT1 could enhance the CSC-like characteristics of colon cells.

Knockdown of SIRT1 significantly decreased the expression of N-cadherin and Vimentin and increased the expression of E-cadherin, whereas overexpression of SIRT1 increased the expression of N-cadherin and Vimentin and decreased the expression of E-cadherin (Fig. 4F). Furthermore, we performed Transwell™ assays to determine the invasion and migration potential of cells with SIRT1 knockdown or overexpression. Compared with those of the control group, the migration and invasion rates of SIRT1 overexpressing cells were higher, while those of SIRT1 knockdown cells were significantly lower (Fig. 4D, E). In brief, SIRT1 could enhance the EMT of colon cells. Additionally, overexpression or knockdown of SIRT1 significantly influenced the expression of the components of the Wnt/β-catenin pathway and PI3K/AKT/mTOR pathway mentioned above (Fig. 4G, H).

**Fig. 5:**
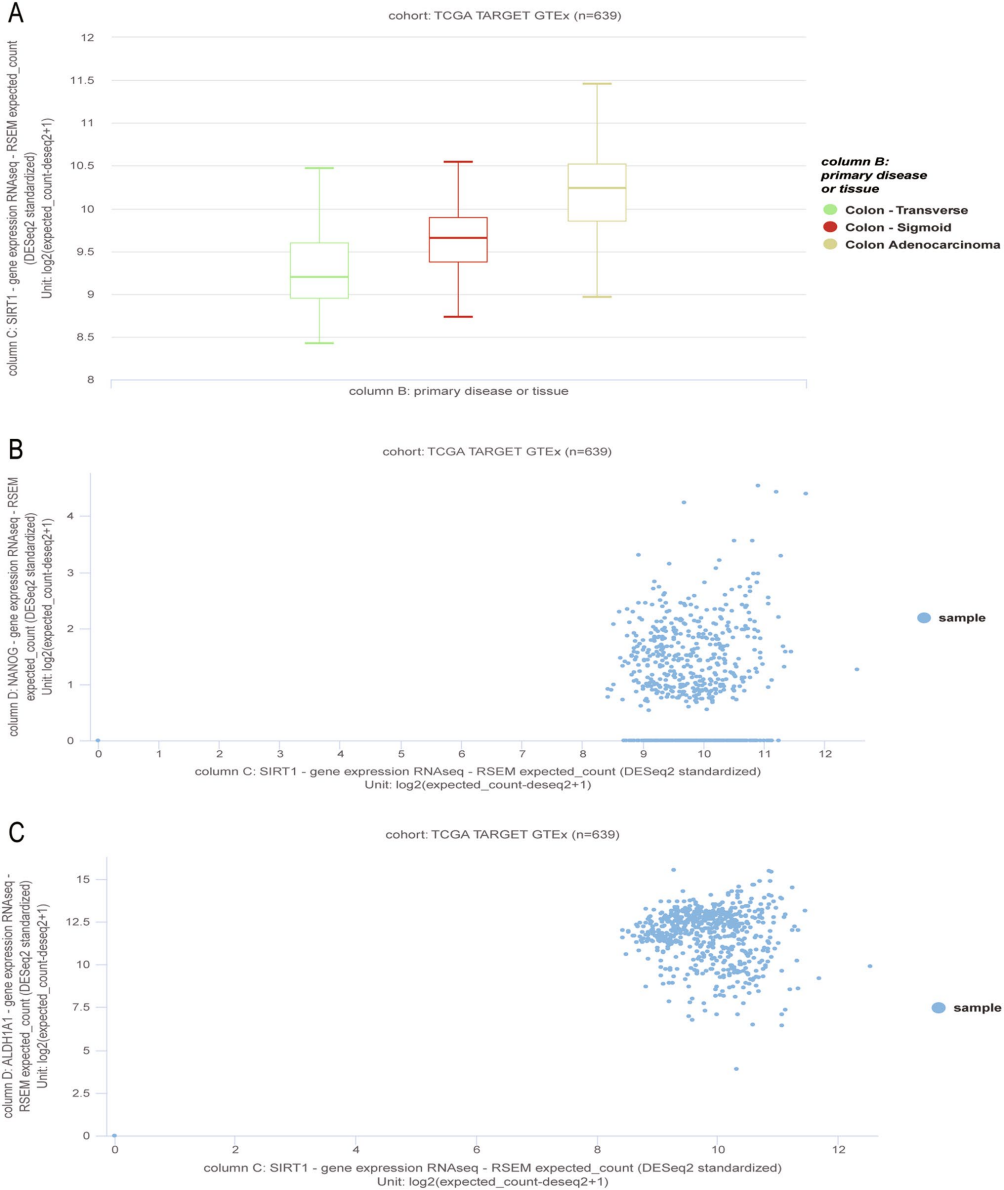
Using UCSC Xena platform to analyze the human colon samples from TCGA GTEx database (n = 639). **A** TCGA GTEx transcriptome analysis of SIRT1 expression in human colorectal tissues (n = 639) by UCSC Xena platform. **B** and **C**. The correlation of NANOG, ALDH1A1 and SIRT1 expression in colorectal samples (n = 639) of TCGA GTEx database were measured by UCSC Xena platform

We further used the UCSC Xena platform to analyze human colon samples from the TCGA GTEx database (n = 639). Based on the transcriptome analysis results, the expression of SIRT1 was upregulated in colon adenocarcinoma samples (Fig. 5A). The results also revealed a positive correlation between SIRT1, Nanog and ALDH1A1 (Fig. 5B, C).

### SIRT1, Wnt/β-catenin signaling pathway and PI3K/AKT/mTOR signaling pathway are critical in propofol-mediated stemness and EMT

**Fig. 6:**
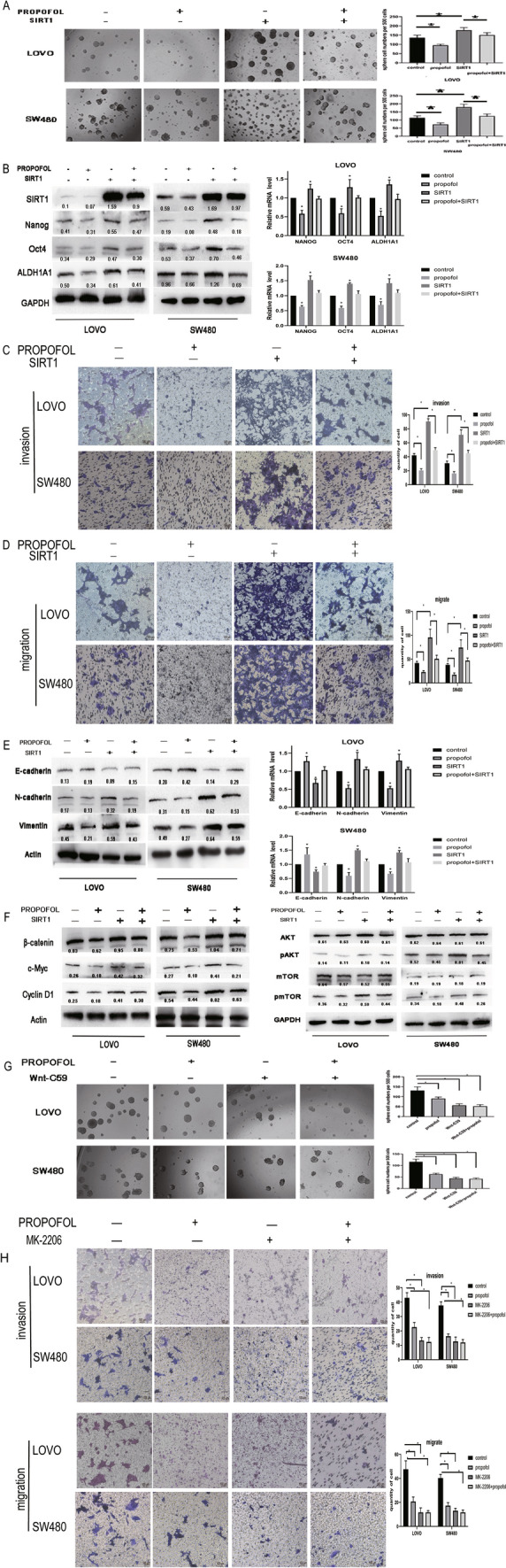
SIRT1 is critical in propofol-mediated stemness and EMT. **A** Analysis of the self-renewal abilities of LOVO and SW480 cells expressing in control, propofol, SIRT1 and SIRT1 + propofol. Scale bar = 100 μm. **B** Expression of stem cell markers in LOVO and SW480 cells was detected by Western blot and qPCR. **C** and **D** The invasion and migration ability of LOVO and SW480 cells were inspected by transwell assays. Scale bar = 100 μm. **E** Expression of EMT markers in LOVO and SW480 cells was detected by western blot and qPCR. **F** Expression of PI3K/AKT/mTOR signaling pathway and Wnt/β-catenin signaling pathway was detected by Western blot. **G** Analysis of the effect of Wnt/β-catenin pathway inhibitor Wnt-C59 on the self-renewal ability of propofol-treated cells and control cells (scale bar, 100 μm). **H** Analysis of the effect of PI3K/AKT/mTOR pathway inhibitor MK-2206 on invasion and migration ability of propofol-treated cells and control cells by Transwell assays. Scale bar = 100 μm *P < 0.05

To further demonstrate that whether the propofol induced changes in stemness and EMT were caused by SIRT1 inhibition, we first overexpressed SIRT1 in LOVO and SW480 cells and then treated them with propofol. We observed that SIRT1 upregulation dramatically enhanced stemness (Fig. 6A, B), EMT (Fig. 6C–E) and pathway component expression (Fig. 6F) compared with the control group. Moreover, after SIRT1 upregulation, propofol treatment did not inhibit stemness (Fig. 6A, B), EMT (Fig. 6C–E), or the Wnt/β-catenin and PI3K/AKT/mTOR pathways (Fig. 6F). This illustrated that upregulating SIRT1 reversed the inhibitory effects of propofol on stemness and EMT mentioned above, and propofol reversed the promoting effect of upregulating SIRT1. To further confirm the participation of Wnt/β-catenin signaling and PI3K/AKT/mTOR signaling, Wnt/β-catenin pathway inhibitor Wnt-C59 (10 μM, MCE, USA) and PI3K/AKT/mTOR pathway inhibitor MK-2206 (5 μM, Beyotime, China) were added to the cells. Wnt-C59 blocked the propofol-induced decrease in sphere formation (Fig. 6G). MK-2206 blocked the propofol-induced decrease of migration and invasion (Fig. 6H). Taken together, these results indicate that SIRT1, Wnt/β-catenin pathway and PI3K/AKT/mTOR pathway are critical to propofol mediated inhibition of colon tumor stemness and EMT in vitro.

## Discussion

Our study is the first to confirm that propofol could inhibit the stemness and EMT of colon cancer cells at least partially through, SIRT1, and the Wnt and PI3K/AKT/mTOR signaling pathways.

Propofol is a widely used anesthetic in cancer resection surgery. Previous studies have suggested that propofol has anti-tumor potential [4], and propofol-based total intravenous anesthesia (TIVA) is generally associated with better overall survival than volatile anesthesia during cancer surgery [5]. Perioperative plasma from patients undergoing tumor resection with propofol TIVA inhibits tumor cell growth in vitro [26]. For instance, some studies have demonstrated that propofol inhibits invasion and promotes apoptosis in colon cancer [27], and inhibits the expression of EMT biomarkers [28]. Propofol inhibits signaling pathways associated with colon cancer cell proliferation, migration, and EMT by regulating miRNAs [29]. Propofol upregulates miR-219-5p and inhibits the a stemness related Wnt/β-catenin pathway and EMT in hepatocellular carcinoma cells [30]. However, some studies have suggested that propofol promotes the migration and invasion of tumor. For instance, propofol may promote tumor metastasis through GABAA R-TRIM21-Src mechanism [31], and clinical concentrations of propofol promote migration and invasion by upregulating SNAI1 in oral squamous cell carcinoma [32]. We speculate that the discrepancies might result from differences in the molecular features of distinct cancers, and we may choose distinct anesthetics individually based on clinical and molecular features. In terms of stemness, propofol reduces the self-renewal ability of breast cancer [12], bladder cancer [33] and acute myeloid leukemia cells [34], but its effects have not been reported in colon cancer. In this study, we found that propofol could inhibit colon cell stem cell-like characteristics and EMT. Moreover, the levels of stem cell markers and mesenchymal markers, such as ALDH1A1, OCT4, Nanog and N-cadherin were decreased in these cells, and the epithelial marker E-cadherin was upregulated. The results of the study support that propofol may have anti-tumor potential and better in surgical treatment of colon cancer, but further investigation is needed. Overall, propofol may be beneficial in patients undergoing colon cancer resection, who express high level of SIRT1.

Propofol inhibits SIRT1 expression in colon cancer. Sirtuins are a highly conserved family of nicotinamide adenine dinucleotide (NAD^+^)-dependent protein lysine modifying enzymes with deacetylase, adenosine diphosphate-ribosyltransferase and other deacylase activities [35]. SIRT1 plays a dual role in cancer promotion and suppression, depending on tissue contexts and the temporal and spatial distribution of SIRT1 upstream and downstream factors [36], whereas SIRT1 is an oncoprotein that promotes the stemness of colorectal cancer cells [3], as well as liver cancer stem cells [37]. We found that overexpression of SIRT1 could enhance LOVO and SW480 stemness and EMT. In contrast, the silencing of SIRT1 by siRNA reduced the frequency and invasion and migration ability of cancer cells. Additionally, as we demonstrated, SIRT1 has been proposed as a key regulator of cancer metastasis that promotes EMT and is involved in various signaling pathways related to carcinogenesis [38]. Taken together, these findings indicate that SIRT1 could regulate the stemness and EMT of colon cancer cells, and that SIRT1 may serve as potential target for colon cancer treatment. For colon cancer patients who express high levels of SIRT1, propofol may be better than other anesthetics.

The interconnection between the Wnt/β-catenin and PI3K/AKT/mTOR pathways has been widely demonstrated in distinct cancer settings [39]. Wnt/β-catenin is a key pathway for regulating stemness [40], which contains extremely complex crosstalk between other signal pathways and plays a pivotal role. The Wnt/β-catenin pathway is upregulated in the majority of colorectal cancers [41], and has been demonstrated to be involved in CSC properties and EMT. For instance, Wnt signaling regulates stem-like properties in human lung adenocarcinoma cell lines [42], and miRNAs alter Wnt/β-catenin signaling in EMT [43]. Drugs of targeting Wnt secretion, and inhibitors of the downstream Wnt pathway are currently undergoing clinical trials [40]. The PI3K/Akt/mTOR pathway plays a fundamental role in the maintenance of stemness, EMT, proliferation, migration, differentiation, autophagy, and upregulation of the mTOR pathway enhances CSC migration and invasion by inducing EMT [44]. For instance, PI3K/AKT/mTOR signaling has a positive correlation with transcriptomic stemness in breast cancer [45]. mTOR suppression decreased ALDH1 activity, a marker of CSCs in colorectal cancers [46]. An mTOR inhibitor decreased the survival and suppressed the invasion of colorectal CSCs in vitro, and suppressed tumor growth in vivo [46]. Many of inhibitors of the pathway are ongoing in clinical trials. Our results showed that propofol decreased the expression of key molecules of the Wnt/β-catenin and PI3K/AKT/mTOR pathways. In addition, the expression of these biomarkers was significantly increased by SIRT1 plasmid DNA and decreased by siSIRT1. The results also showed that the addition of Wnt-C59 and MK-2206 blocked the propofol induced decrease in sphere-forming and invasion-migration, further supporting the role of the Wnt/β-catenin pathway and PI3K/AKT/mTOR pathway as targeted downstream signaling of propofol. These data suggest that the Wnt/β-catenin and PI3K/AKT/mTOR pathways contribute to the promotion of colon cancer stemness and EMT.

A limitation of our study is that the exact mechanism by which propofol interacts with SIRT1 was not explored, and we were unable to assess clinical tissue. We did not establish a metastasis model to further investigate whether propofol affects colon cell metastasis in vivo; therefore, we will further examine the antimetastatic activity of propofol in mice intravenously administered colon cancer cells. Propofol binding other receptors or oncoproteins may be explored in the future.

## Conclusions

In conclusion, we demonstrated that propofol inhibits the stemness and EMT of colon cancer cells by downregulating the SIRT1 and the Wnt/β-catenin and AKT/mTOR signaling pathways. These effects are reversed after SIRT1 overexpression, suggesting a SIRT1-mediated mechanism. The results indicate that propofol may be beneficial in patients with high SIRT1 expression undergoing colon cancer resection.

## Supplementary Information

**Additional file 1.**

## Data Availability

The data that support the findings of this study are available from the corresponding author upon reasonable request.
