# Supplementary material for: Propofol inhibits colon cancer cell stemness and epithelial-mesenchymal transition by regulating SIRT1, Wnt/β-catenin and PI3K/AKT/mTOR signaling pathways
Source: Discov Oncol. 2023 Jul 25;14:137. doi: 10.1007/s12672-023-00734-y (PMC10368601; doi:10.1007/s12672-023-00734-y)
Supplement: Supplementary file 1 — Additional file 1. [file 12672_2023_734_MOESM1_ESM.doc]

supplement document

| antibody | Cat No. |
| --- | --- |
| SIRT1 | sc-74504, Santa Cruz |
| SIRT1 | 13161-1-AP, Proteintech |
| ALDH1A1 | 15910-1-AP, Proteintech |
| ALDH1A1 | 36671S, CST |
| vimentin | 60330-1-Ig, Proteintech |
| Nanog | 4903, CST |
| Oct4 | 2750, CST |
| β-catenin | 9582, CST |
| E-cadherin | 3195, CST |
| N-cadherin | 4061, CST |
| c-Myc | 5605, CST |
| p—AKT | 4060, CST |
| AKT | 2920S, CST |
| mTOR | 2983S, CST |
| p-mTOR | 5536S, CST |
| α-Tubulin | 11224-1-AP, Proteintech |
| actin | abs830031, Absin |
| GAPDH | 10494-1-AP, Proteintech |
| Cyclin D1 | 2978, CST |
| Ki67 | GB111141, Servicebio |
| CD44 | GB112054, Servicebio |
| N-cadherin | GB111273, Servicebio |

Sequences of PCR primers

| H-SIRT1-F | CCAAACTTTGCTGTAACCCTGT |
| --- | --- |
| H-SIRT1-R | TCATCATTACTCTTAGCTGCTTGG |
|  |  |
| H-GAPDH-F | GGAAGCTTGTCATCAATGGAAATC |
| H-GAPDH-R | TGATGACCCTTTTGGCTCCC |
|  |  |
| H-NANOG(1)-F | CCAAAGGCAAACAACCCACT |
| H-NANOG(1)-R | GTCACACCATTGCTATTCTTCGG |
|  |  |
| H-oct4-F | TCTATTTGGGAAGGTATTCAGCC |
| H-oct4-R | CCTCTCACTCGGTTCTCGATACTG |
|  |  |
| H-ALDH1A1-F | CTTACCTGTCCTACTCACCGATTT |
| H-ALDH1A1-R | TGCCTTGTCAACATCCTCCTTA |
|  |  |
| H-E-cadherin-F | GAGAACGCATTGCCACATACAC |
| H-E-cadherin-R | GAGCACCTTCCATGACAGACCC |
|  |  |
| H-N-Cadherin-F | AAGAGGCAGAGACTTGCGAAAC |
| H-N-Cadherin-R | TGGAGTCACACTGGCAAACCTT |
|  |  |
| H-vimentin（1）-F | ATCTGGATTCACTCCCTCTGGTT |
| H-vimentin（1）-R | CGTGATGCTGAGAAGTTTCGTTG |
